# Three-dimensional C-scan-based generation adversarial network with synthetic input to improve optical coherence tomography angiography

**DOI:** 10.1117/1.JBO.30.5.056006

**Published:** 2025-05-09

**Authors:** Jingjiang Xu, Zhongwu Feng, Haixia Qiu, Peijun Tang, Kai Gao, Yanping Huang, Gongpu Lan, Jia Qin, Lin An, Gangyong Jia, Qing Wu

**Affiliations:** a Foshan University, Guangdong-Hong Kong-Macao Intelligent Micro-Nano Optoelectronic Technology Joint Laboratory, Foshan, China; b Guangdong Weiren Meditech Co., Ltd, Innovation and Entrepreneurship Teams Project of Guangdong Pearl River Talents Program, Foshan, China; c Foshan University, School of Mechatronic Engineering and Automation, Foshan, China; d Chinese PLA General Hospital, The First Medical Centre, Department of Laser Medicine, Beijing, China; e South China Normal University, College of Biophotonics, Guangzhou, China; f Sun Yat-sen University, Guangdong Provincial Key Laboratory of Ophthalmology and Visual Science, State Key Laboratory of Ophthalmology, Guangdong Provincial Clinical Research Center for Ocular Diseases, Zhongshan Ophthalmic Center, Guangzhou, China; g Hangzhou Dianzi University, School of Computer Science, Hangzhou, China

**Keywords:** optical coherence tomography angiography, deep learning, image enhancement, image synthesis, dermatology

## Abstract

**Significance:**

Optical coherence tomography angiography (OCTA) usually suffers from the inherent random fluctuations of noise and speckles in the imaging system. Previous deep learning methods have mainly focused on improving the quality of B-scan blood flow images or *en face* projection images. We propose a deep learning method to reconstruct high-quality 3D vasculature, which fully utilizes the volumetric OCTA data and the topological features of the vascular network.

**Aim:**

We propose a deep learning method called the three-dimensional C-scan-based generation adversarial network (3DCS-GAN) to improve vascular visualization for volumetric OCTA data.

**Approach:**

To train the network, we superimposed the single-shot *en face* OCTA images on avascular noisy C-scan images to synthesize the input data and used the multiple averaged *en face* OCTA images as the reference labels. The deep learning algorithm is based on Pix2Pix architecture and consists of a generator model and a discriminator model. A perceptual loss function was utilized by combining content loss and adversarial loss. The proposed algorithm is applied to the C-scan images depth-by-depth to suppress the background noise and enhance vascular visualization in the 3D OCTA data.

**Results:**

The proposed method has improved the contrast-to-noise ratio of cross-sectional OCTA images by ∼2 times. It greatly enhances the visualization of blood vessels in the deep layer and offers much clearer blood vessel topology in the 3D volume-rendering of OCTA. 3DCS-GAN has exhibited superior image enhancement compared with alternative methods. It has been used to enhance the OCTA images of port wine stain disease for clinical investigation.

**Conclusions:**

It demonstrates that the proposed 3DCS-GAN can greatly improve vascular visualization in the deep layer, provide better image quality than the multiple averaged OCTA images, and achieve superior image enhancement for volumetric OCTA data.

## 1 Introduction

Optical coherence tomography (OCT) is a fast, noninvasive, high-resolution optical imaging technique for cross-sectional and volumetric imaging by detecting the interferometric signal to measure the echo delay of backscattered light. Since its first introduction by Huang et al.,^1^ it has become an indispensable imaging tool for diagnosing and evaluating many eye diseases in the field of ophthalmology.^2^^–^^5^ In recent years, research in dermatology and cardiovascular fields has also received increasing attention.^6^^–^^9^ With the development of OCT technology in recent decades, various functional imaging methods, such as optical coherence tomography angiography (OCTA), polarization-sensitive OCT (PS-OCT), and optical coherence elastography, have been gradually developed.^10^^–^^15^ Among them, OCTA is a microvascular imaging tool with three-dimensional visualization and high spatial resolution that does not require exogenous contrast agents. The blood flow image is obtained by capturing the dynamic signal among multiple scans at the same imaging position. This functional imaging modality has been widely used in preclinical procedures and clinical diagnoses. Several algorithms can be used for OCTA to extract blood flow signals, such as the phase-based variance method,^16^ the split-spectrum amplitude-decorrelation angiography method,^17^ and the complex-based optical micro-angiography (OMAG) method.^18^ However, the functional imaging modality of OCTA suffers from random fluctuations of noise and speckles inherent in the imaging system.^19^ These undesirable artifacts can generate a false appearance of blood flow and may lead to incorrect interpretation and quantification of OCTA images.

To improve the quality of OCTA images, many methods have been developed to reduce these noises while ensuring the true blood flow signal remains unchanged. Repeated B-scans of the same location with multiple *en face* image averaging were used to achieve noise reduction and enhance the visualization of blood vessels to improve quantitative assessment.^20^^,^^21^ In the field of OCT or OCTA, a B-scan is a cross-sectional view, showing depth-resolved structural or vascular information in a vertical slice. A C-scan image is a top-down view of a specific tissue layer, displaying lateral structural or vascular details at a chosen depth. An *en face* image is generated by projecting data from multiple C-scans to create a top-down map, commonly used to laterally visualize the structural topology or vascular networks. However, the acquisition time to obtain more data and improve the image quality was longer. In clinical practice, this method may have more motion artifacts due to the involuntary movements of patients during the long acquisition time. Moreover, the motion drift and distortion among multiple *en face* acquisitions require complex registration operations before averaging, further increasing the workload. Numerical filtrations such as median filtering, Gabor filtering,^22^ and Hessian filtering^23^ are considered efficient approaches to smooth the OCTA images and improve image quality. Chlebiej et al.^24^ developed an elliptical directional filtering method and used the best matching ellipses for median filtering of the C-scans, which reduced the granular noise and increased the continuity of vessels. However, these filtering-based methods somehow weaken the high intensity of vascular signals and amplify noise to a certain degree which may be mistakenly considered blood vessels.

The recent significant advancements in artificial intelligence, particularly in image processing with deep learning, have greatly benefited the field of medical diagnosis and become a major driving force behind the development of various deep learning techniques.^25^^–^^29^ The image enhancement for OCTA by deep learning is gaining more and more attention. Lee et al.^30^ reconstructed the cross-sectional retinal OCTA images by training a U-shaped autoencoder network to recognize the blood flow signals from individual structural B-scan images. Later, a deep learning pipeline was proposed to improve the image quality of cross-sectional OCTA images with better SNR and speckle variance eliminating from several consecutive B-scans, which demonstrated the superiority of the deep learning–based pipeline over the traditional OCTA algorithms.^31^ Jiang et al.^32^ conducted a comparative study of four representative network architectures to reconstruct high-quality cross-sectional angiograms. They found that U-shaped and multipath models were two suitable architectures that can enhance OCTA images. In 2020, a cycle-consistent adversarial network was developed to enhance the digital resolution of low transverse sampling OCTA images in the *en face* plane.^33^ A deep learning–based high-resolution angiography reconstruction network was introduced to reconstruct enhanced $6\times 6\;{\mathrm{mm}}^{2}$ *en face* OCTA images in a superficial vascular complex slab with significantly lower noise intensity, stronger contrast, and better vascular connectivity than original angiograms.^34^ Kadomoto et al.^35^ applied a U-Net-based deep learning denoising method to process the *en face* OCTA images of patients with retinal diseases, where the algorithm removed the background noise and smoothed the rough vascular surfaces, which made a significant difference in quantitative vascular measurements. Kim et al.^36^ proposed an integrated end-to-end deep neural network framework with a two-staged adversarial training scheme to simultaneously enhance the sampling resolution and blood flow quality for *en face* OCTA images with undersampled data and fewer repeated B-scans, which could accelerate the OCTA imaging speed and also improve the image quality.

However, many previous studies first used deep learning methods to enhance the B-scan OCTA images and then converted the enhanced 3D volumetric OCTA data to *en face* angiogram by maximum intensity projection (MIP)^37^ for better vascular visualization. However, the B-scan-based approaches typically require tens of repeated B-scans (each taking ∼10  ms) to obtain high-quality ground truth data for network training, resulting in a prolonged data acquisition time for a 3D OCTA volume, which may exceed several minutes.^31^ Moreover, the blood vessels in the B-scan view are relatively independent, and the deep learning methods might not correctly extract the vascular network features and might generate false blood flow signals. Other studies on deep learning–based OCTA image enhancement applied their algorithms to the *en face* OCTA images.^33^^–^^35^ Because the OCTA images in the axial direction are strongly affected by shadowing artifacts and the blood vessels are primarily oriented laterally, the *en face* OCTA images are preferable for intuitive interpretation of the vascular structure. However, these *en face* view–based methods ignored the depth information of blood vessels and did not fully utilize the 3D vascular network topology to visualize volumetric vasculature better.

In this regard, we propose a novel deep learning approach to achieve superior volumetric OCTA image enhancement. A generative adversarial network (GAN) is employed to learn the topological feature of OCTA image in *en face* view. Unlike previous studies, our deep learning algorithm is applied to sectioned vascular images in the C-scan plane to reconstruct high-quality OCTA images depth-by-depth. The input data are synthesized by combining the *en face* projection of OCTA images with the corresponding C-scan noisy images at a superficial depth. Finally, we compared the quantitative and qualitative results with existing image enhancement methods. Meanwhile, we also tested and quantitatively analyzed the OCTA images of port wine stain (PWS) disease using the enhancement method proposed in this article. The results show that this enhancement method can significantly improve the continuity of vascular structures in OCTA images while effectively reducing noise interference.

## 2 Materials and Methods

**Fig. 1:**
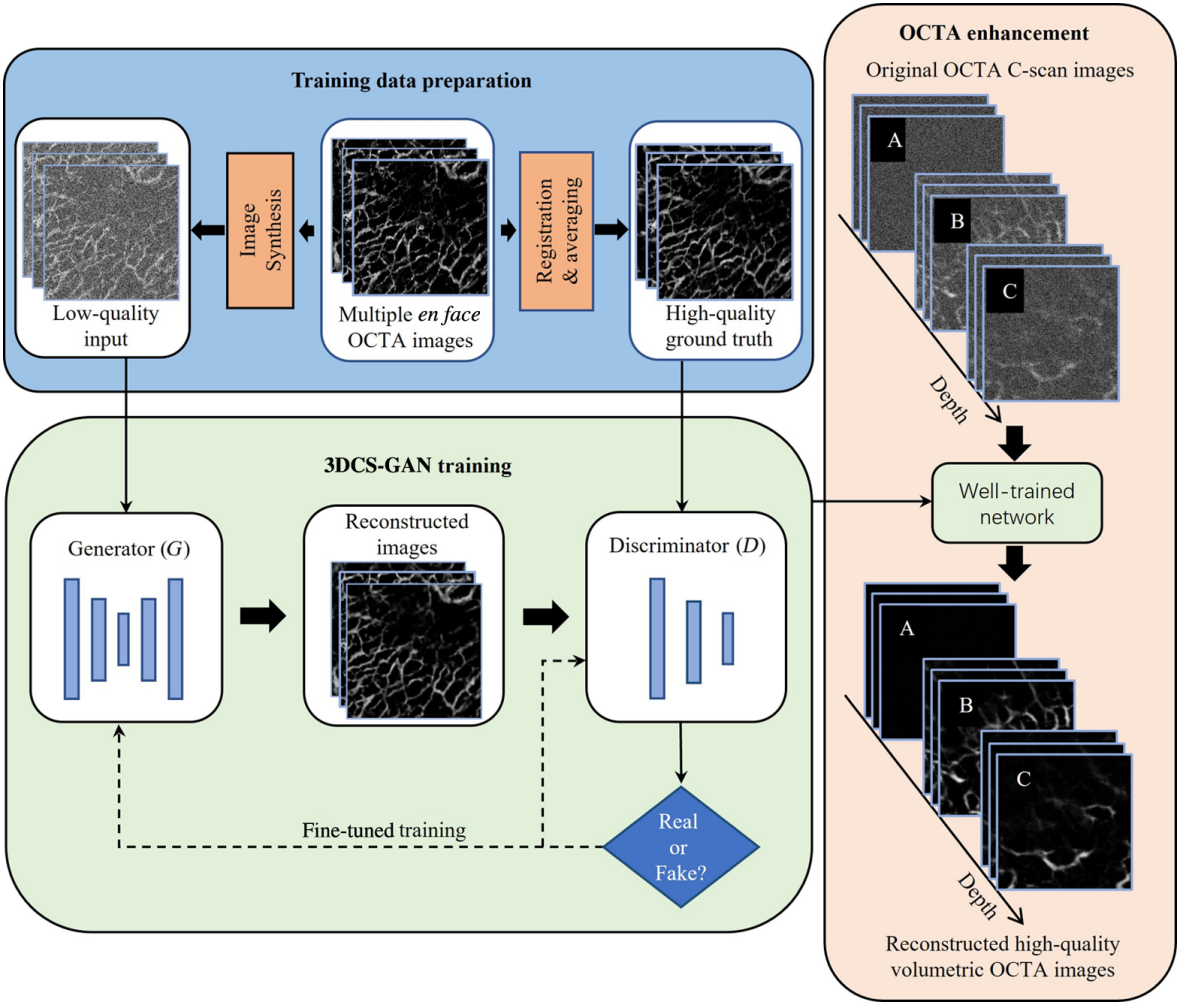
Schematic diagram of our 3DCS-GAN with synthetic input.

Figure 1 shows the schematic diagram of our deep learning method for the quality enhancement of OCTA images. Multiple *en face* OCTA images were utilized for the training data preparation to generate high-quality reference labels by registration and averaging. These *en face* images were superimposed on noisy images to synthesize low-quality input. We designed a GAN-based model and iteratively optimized the network architecture and hyperparameters to maximize enhancement performance. For OCTA image enhancement, the well-trained network is applied to original OCTA C-scan images to reconstruct high-quality volumetric OCTA images layer by layer. We conducted qualitative and quantitative comparisons and analyses for the reconstructed images to verify the superiority of this method for OCTA image enhancement.

### 2.1 OCT System and Data Acquisition

In this study, the OCTA data were collected by a home-built swept-source OCT system and processed using the OMAG algorithm, which has been described in our previous study.^38^ The system uses a tunable vertical cavity surface emitting laser (MEMS-VCSEL) working at 1060 nm with a 200 kHz sweep rate. A fiber-based interferometer that delivered ∼5  mW optical power in the sample arm was built. The sensitivity is ∼110  dB, whereas the axial resolution is 10  μm, and the lateral resolution is 18  μm. The OCT system was designed for skin imaging with a compact scanning probe and a sample stage for stabilizing the hand. Twenty healthy volunteers aged between 20 and 30 were enrolled in the study. We collected the OCTA data by imaging the thenar area of the palm in both hands. Ten volumes of OCTA data were consecutively acquired on the same thenar area of the palm, which generated 400 volumes of OCTA data in total to reconstruct high-quality OCTA images as reference labels. Each volume of OCTA data consisted of 300 A-lines in a fast direction and 300 B-scan locations in a slow scanning direction with four repeated B-scans, which took 4 s for data acquisition.

### 2.2 Data Preparation

**Fig. 2:**
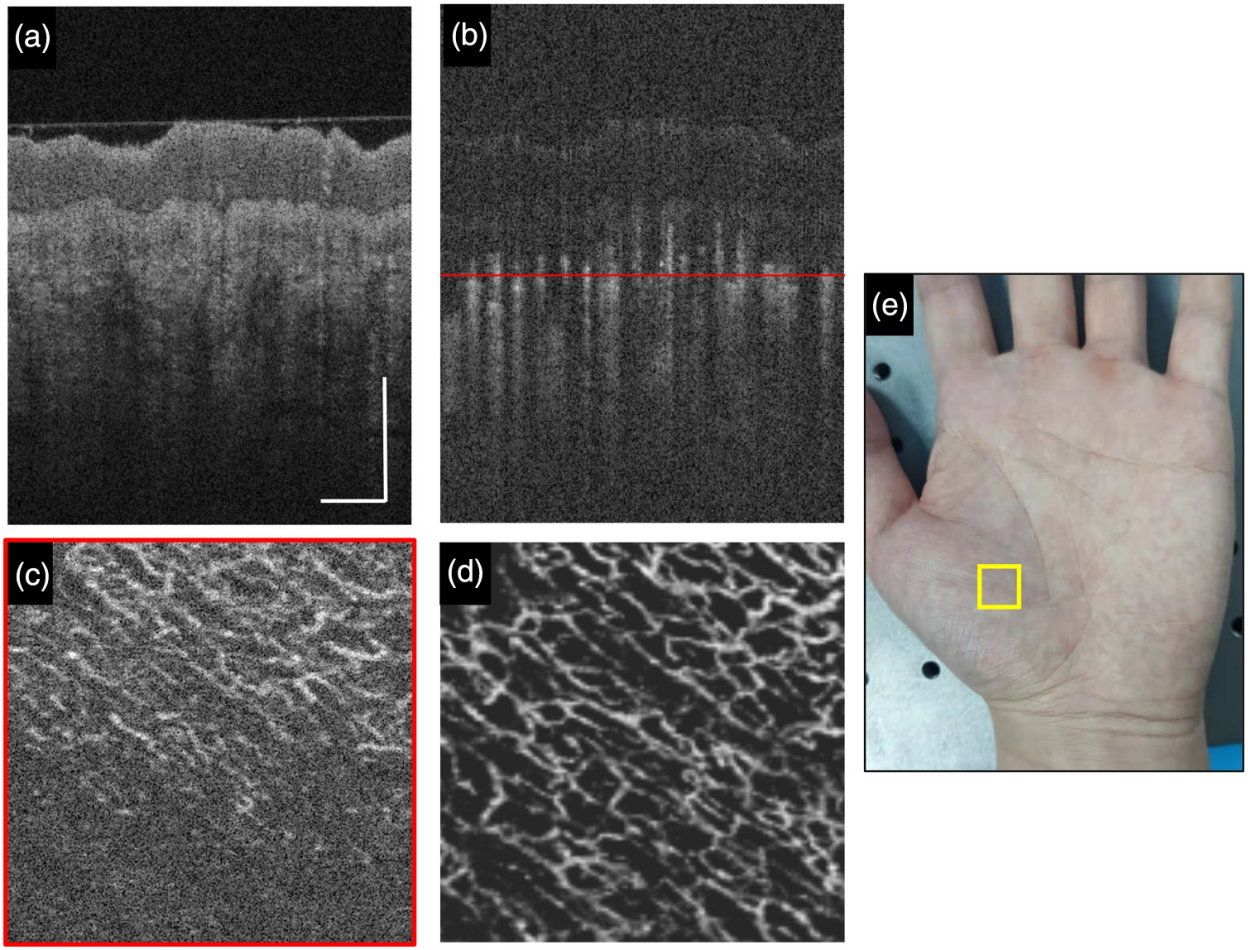
(a) Representative B-scan structural OCT image. (b) The corresponding B-scan blood flow OCT image. (c) Sectioned C-scan OCTA image at the depth position indicated by the red line in (b). (d) *En face* projection of OCTA image. (e) Photograph of a human hand for OCTA imaging, where the yellow square shows the imaging area. The white scale bar is 0.5 mm. The field of view (FOV) is $3\times 3\;{\mathrm{mm}}^{2}$.

The representative OCT structure vasculature images and photographs of the hand as the imaging subject are shown in Fig. 2. The cross-sectional images, as shown in Figs. 2(a) and 2(b), provide depth-resolved visualization of the structure and blood flow in the thenar skin of the palm. Figure 2(c) shows a sectioned C-scan OCTA image at the depth position indicated by the red line in Fig. 2(b). The sectioned C-scan OCTA image shows part of the cutaneous vasculature, which has a large amount of speckle noise and breaks branches in the vascular network. Figure 2(d) is the *en face* OCTA image by MIP from stacks of C-scans with the fine tuning of the brightness and contrast. The *en face* MIP greatly reduces the speckle noise and demonstrates a more comprehensive visualization of the cutaneous vasculature.

#### 2.2.1 Registration and averaging for reference labels

**Fig. 3:**
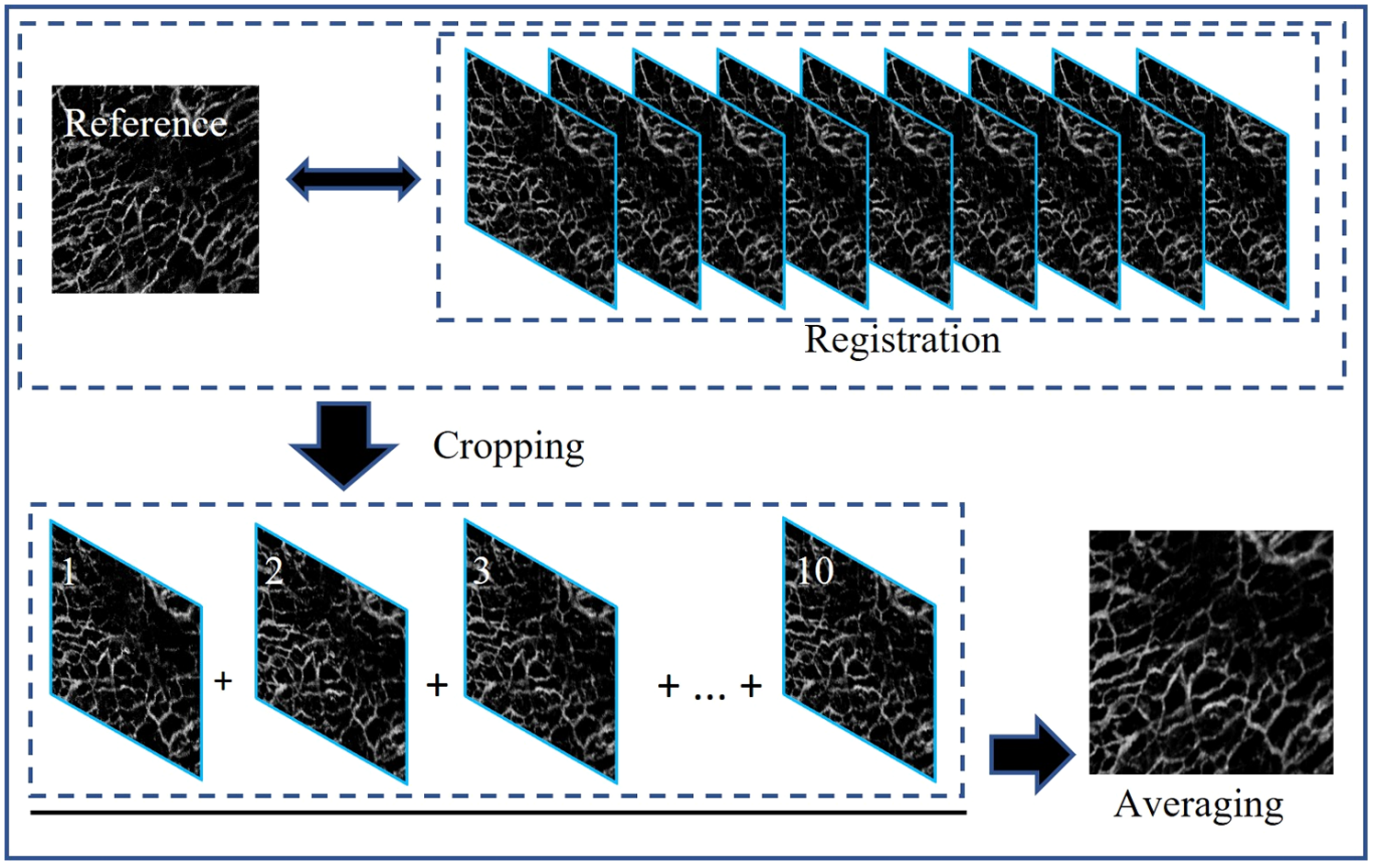
Workflow chart of registration and averaging among multiple OCTA images.

**Fig. 4:**
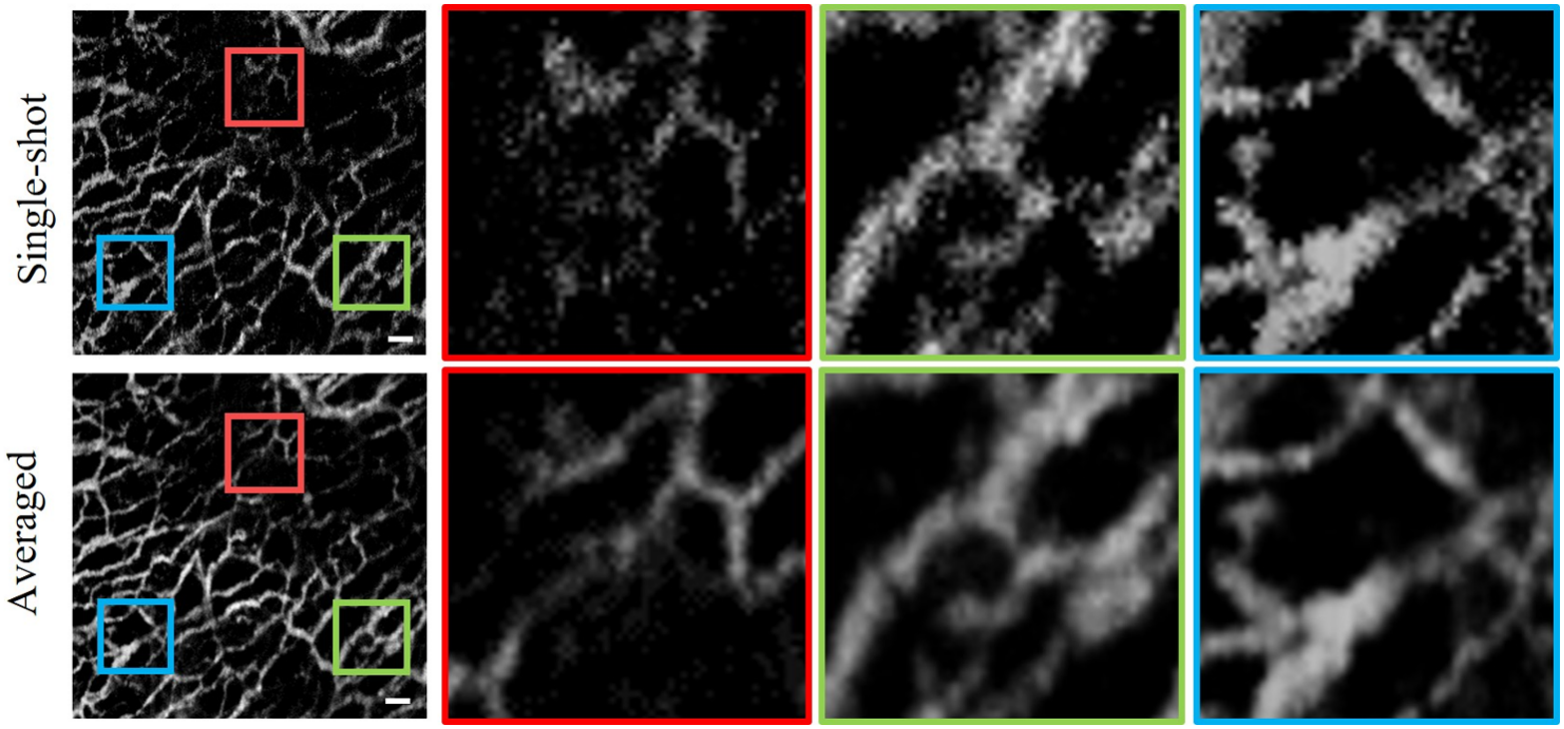
Visual comparison of single-shot *en face* OCTA image and averaged *en face* OCTA image with registration. The scale bar is 250  μm.

As shown in Fig. 2(d), the quality of single-shot *en face* OCTA images is still not good enough because the blood vessels have many granular points and are not well connected. The deep learning method requires specific data preparation for network training to achieve superior image enhancement for OCTA. We consecutively acquired 10 volumes of OCTA data on the same area of the skin and performed registration and averaging on these *en face* images to obtain high-quality OCTA images. Figure 3 shows the registration and averaging workflow chart among multiple OCTA images. Because the data acquisition time of 10 OCTA volumes was relatively long, motion drift and tissue distortion among these OCTA volumes could be observed. Before averaging, we used the image registration method based on the scale-invariant feature transform algorithm.^39^ One of the 10 *en face* OCTA images is selected as a reference, and the other 9 are registered to match the reference. The unmatched peripheral areas are cut off, and all that remains are the well-registered *en face* images with 256×256  pixels per image. Finally, we performed averaging operation on the 10 cropped images to obtain a cleaner and smoother image. The visual comparison of single-shot *en face* OCTA and averaged *en face* OCTA is shown in Fig. 4. The zoom-in views demonstrate that registration and averaging operation of multiple OCTA images greatly reduce the granular noise and enhance visualization of blood vessels in the skin. The high-quality *en face* OCTA images generated by registration and averaging can serve as ground truth labels to train our deep learning network.

#### 2.2.2 Low-quality input image synthesis

**Fig. 5:**
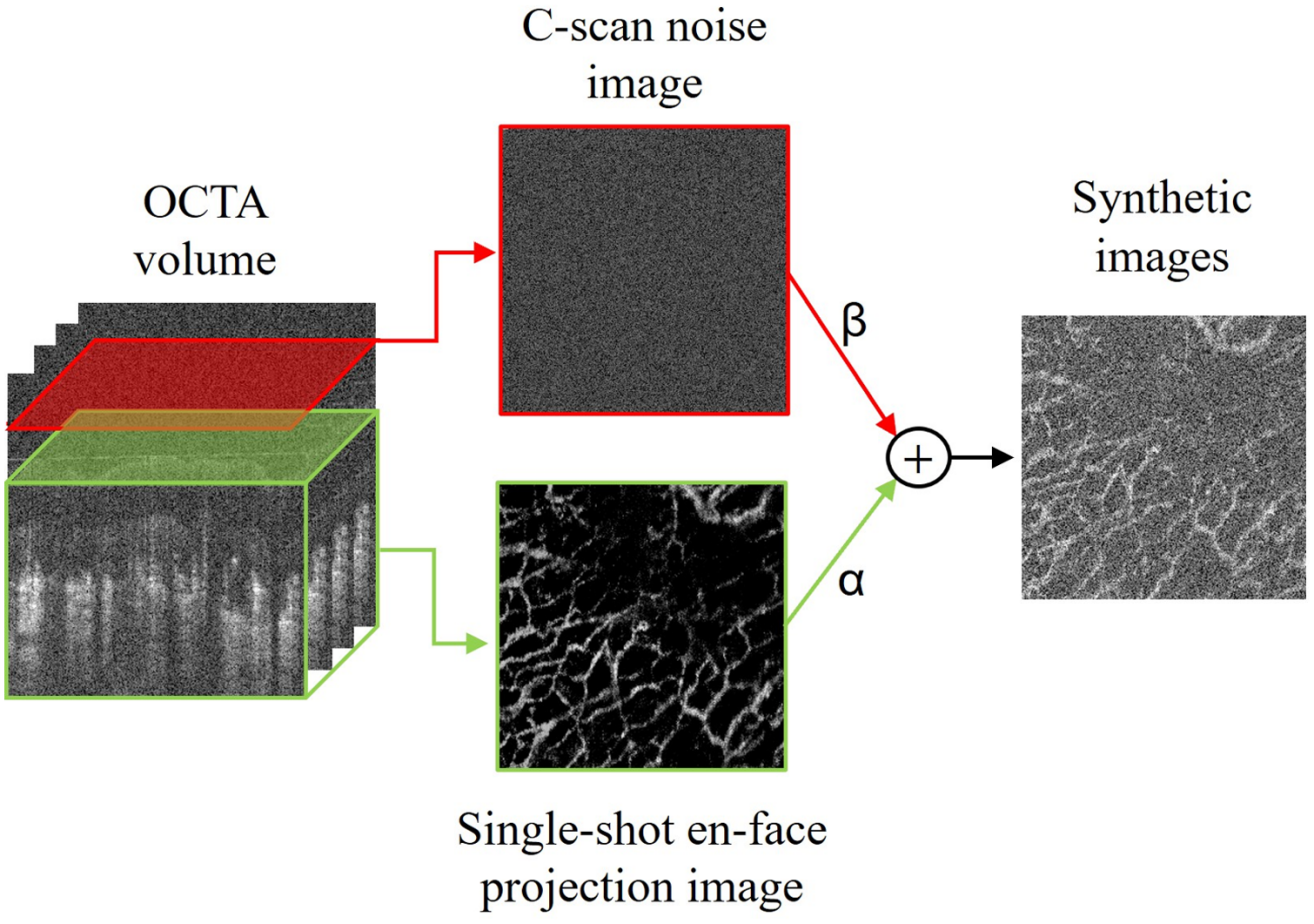
Process flowchart of input image synthesis. Overlay a single C-scan noise image without vessel information with a single *en face* image. The resulting composite image serves as input, replacing the single C-scan.

We are supposed to apply our deep learning algorithms to each sectioned C-scan OCTA image at different depths, but obtaining high-quality C-scan OCTA images as reference labels for network training is difficult. Image synthesis is an efficient alternative to obtaining proper deep learning training samples. Here, we add noise to a single *en face* projection image to synthesize the input image, which has similar noise characteristics and vascular patterns to a single C-scan image. Therefore, the *en face* OCTA image obtained through registration and averaging can be considered the reference labels for the synthetic image. The flowchart of the input image synthesis process is shown in Fig. 5.

We first define a single-shot *en face* projection image as E. A sectioned C-scan noisy image at a superficial depth position without blood vessels was selected and defined as C. The synthesized image S is as follows:

S=norm(α×E+β×C), (1)

where α and β are the weights of E and C, respectively, and the symbol of *norm* refers to the operation of normalization. The values of the weights will be discussed in Sec. 3.

There are 400 sets of OCTA data in the study, where we synthesize 350 sets of *en face* images by the same operation for network training, and the remaining 50 sets of data are used as the test set. We added 20 noisy images without vascular information and 20 completely black images to the training and label sets. There is no validation data set due to the limited training data. The images were also rotated and cropped for data augmentation in each epoch to prevent network overfitting.

### 2.3 Network Model

**Fig. 6:**
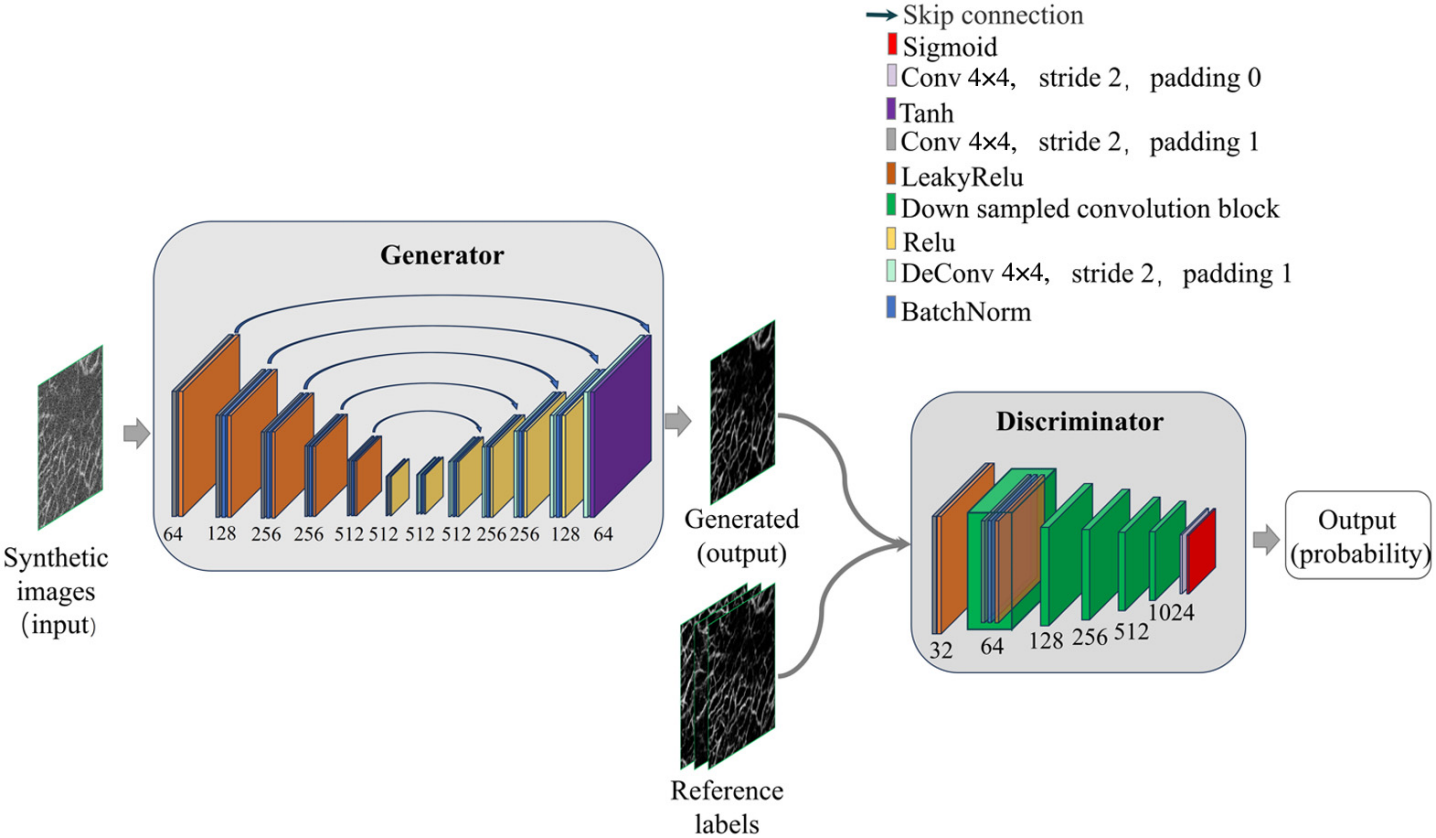
Schematic of 3DCS-GAN network model.

GAN has been commonly used in image processing. The adversarial training based on a minimum–maximum game in GAN can improve the visual quality of reconstructed images very close to real high-quality images. The denoising deep learning algorithm we used is based on Pix2Pix architecture^40^ and consists of a generator model (G) and a discriminator model (D) (shown in Fig. 6). The generator uses a U-shaped (encoder–decoder) network structure to extract features from the image through the downsampling part and then reconstructs the image with the extracted features through the upsampling part. Each convolutional block in the downsampling part consists of a convolutional layer (a convolutional kernel with a convolutional structure of 4×4, a step size of 2, and a padding of 1), a BatchNorm, and a LeakyRelu layer. In each upsampling part, the convolutional block consists of a transposed convolutional layer (the convolutional structure is a 4 × 4 convolutional kernel, step size is 2, and padding is 1), a BatchNorm, and a Relu layer. In addition, a tanh layer is added at the end of the structure. The skip connection structure in the middle can introduce the feature information of different scales in the downsampling process into the upsampling process so that the reconstructed image has a better effect. The role of the discriminator is to optimize the training results of the network model. It first consists of a convolutional layer (a convolutional structure with a 4×4 convolutional kernel, step size of 2, and padding of 1) and a LeakyRelu layer. Then, there are five convolutional blocks (same as in the downsampling part), followed by a convolution layer (convolution structure is 4×4 convolution kernel, step size is 2, padding is 0). The final convolutional layer is followed by a sigmoid activation function to output the probability of the input being real.

### 2.4 Loss Function

In OCTA image denoising, we combine the content loss and adversarial loss to form a perceptual loss function as the total generator loss (G_loss)

G_loss=λ×Content_loss+Adversarial_loss, (2)

where λ is the weight, which we set as $10^{3}$. L1 loss is selected as the content loss function in G_loss and is used to minimize the sum of the absolute values of the differences between the real $\left(I_{\text{label}}(i,j)\right)$ and the predicted value ($G(I_{\text{input}}(i,j)))$. The Content_loss is defined as follows:

$$\text{Content}\_\text{loss}=\frac{1}{h\times w}\overset{h}{\underset{i=1}{\sum }}\overset{w}{\underset{j=1}{\sum }}|I_{\text{label}}(i,j)-G(I_{\text{input}}(i,j))|,$$ (3)

where $I_{\text{label}}(i,j)$ represents the high-quality image, $I_{\text{input}}(i,j)$ represents the synthetic input image with noise, (i,j) represents the pixel position of the *en face* image, h and w represent the height and width of the image, respectively, and $G(I_{\text{input}}(i,j))$ represents the image generated by the generator.

The adversarial loss is calculated as follows:

$$\text{Adversarial}\_\text{loss}=E_{I_{\text{input}}}[\log(1-D(G(I_{\text{input}}))].$$ (4)

The discriminator’s loss function (D_loss) is defined as binary cross-entropy, with the objective of distinguishing between real labeled images ($I_{\text{label}}$) and generated images ($I_{\text{input}}$)

$$D\_\text{loss}=-E_{I_{\text{label}}}[\log\;D(I_{\text{label}})]-E_{I_{\text{input}}}[\log(1-D(G(I_{\text{input}})))],$$ (5)

where E represents the mathematical expectation, $D(I_{\text{label}})$ represents the output probability of the discriminator for the real label image, and $D(G(I_{\text{input}}))$ represents the output probability of the discriminator for the generated image.

### 2.5 Evaluation Metrics

We utilize several evaluation metrics to compare the quality of different OCTA images and evaluate the performance of our image enhancement method. Contrast-to-noise ratio (CNR) is a commonly used indicator that objectively evaluates OCTA image quality. The term CNR was defined previously in Ref. 41 using the following equation:

$$\mathrm{CNR}=\frac{\mu_{s}-\mu_{b}}{\sqrt{\sigma_{s}^{2}+\sigma_{b}^{2}}},$$ (6)

where $\mu_{s}$ and $\sigma_{s}$ are the mean and standard deviations of the *en face* OCTA image in the signal region, respectively, and $\mu_{b}$ and $\sigma_{b}$ are the mean and variance of the *en face* OCTA image in the background region, respectively. In this study, we binarized the *en face* OCTA image using a global adaptive threshold method.^42^ The white pixels with a value of 1 in the binarized image represent the signal region of blood flow, whereas the rest of the black pixels with a value of 0 represent the background region.

Vessel density (VD),^23^ vessel diameter index (VDI),^23^ and vascular continuity (VC)^41^ are also commonly used quantitative evaluation indicators of blood vessels. VD is obtained by calculating the ratio of the OCTA signal (white pixels) in the binarized image to the total pixels of the image, and its equation is as follows:

$$\mathrm{VD}=\frac{\sum_{\left(i=1,j=1\right)}^{n}B_{\left(i,j\right)}}{\sum_{\left(i=1,j=1\right)}^{n}X_{\left(i,j\right)}},$$ (7)

where $B_{\left(i,j\right)}$ represents the pixels of the blood flow signal in the binarized image and $X_{\left(i,j\right)}$ represents all pixels in the binarized image. VDI, which represents the average vessel caliber, was calculated by dividing the total vessel area in the binarized image by the total vessel length in the skeleton image as follows:

$$\mathrm{VDI}=\frac{\sum_{\left(i=1,j=1\right)}^{n}B_{\left(i,j\right)}}{\sum_{\left(i=1,j=1\right)}^{n}S_{\left(i,j\right)}},$$ (8)

where $S_{\left(i,j\right)}$ represents the pixel of the blood vessel in the skeletonized image. In addition, we define that the connected blood vessels have at least five continuous pixels in the skeletonized map. VC is defined as the ratio of connected blood vessels to the total blood vessels in the skeletonized image as follows:

$$\mathrm{VC}=\frac{\overset{n}{\underset{\left(i=1,j=1\right)}{\sum }}L_{\left(i,j\right)}}{\overset{n}{\underset{\left(i=1,j=1\right)}{\sum }}S_{\left(i,j\right)}},$$ (9)

where $L_{\left(i,j\right)}$ is the pixel of continuous blood vessels in the skeletonized image. When the value of VC is closer to 1, it means that the image continuity is better.

### 2.6 Implementation

Our model is implemented based on the deep learning framework Python under the Windows 10 operating system and trained by NVIDIA GeForce GTX 1070 graphics processing unit (GPU) with 16 GB random access memory (RAM). The generator and discriminator are optimized using the Adam optimizer with a learning rate of $1\times 10^{-3}$ and batch size of 1 and 120 epochs. It took about 4 h for each training. The average time for processing a C-scan image is 0.0029 s. The total image processing time for each OCTA volume depends on the number of C-scan images. In this study, we usually processed 300 C-scan images, which took 0.87 s for each OCTA volume.

## 3 Results

### 3.1 Selection of Weights

**Fig. 7:**
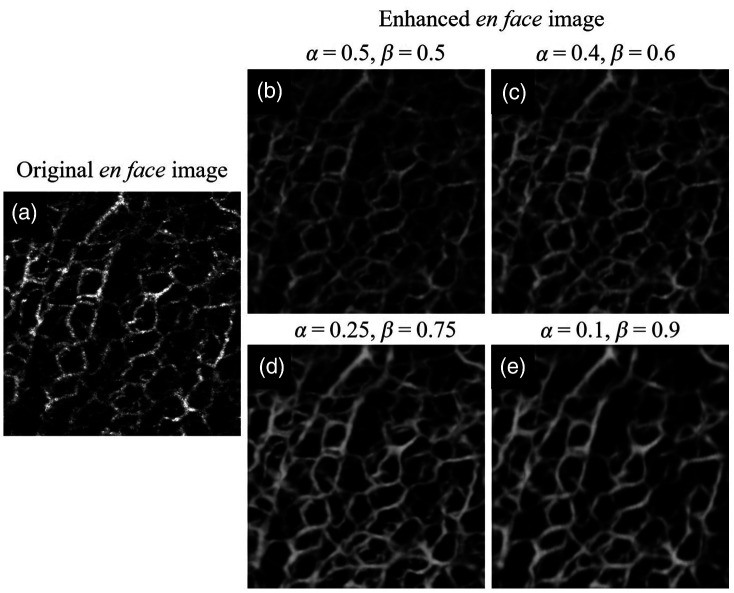
Comparison of OCTA image enhancement with different weights. (a) Original *en face* image. (b)–(e) Enhanced *en face* images with different values of α (i.e., the weight of single *en face* image) and β (i.e., the weight of C-scan noise image).

As described in Sec. 2.2.2, we proposed synthesizing low-quality images with weights of single *en face* and C-scan noise images. After establishing the deep learning framework, we compared multiple enhanced images with different values of α and β to select proper weights for synthesizing training input data. We tried four sets of α and β for network training, and the results are shown in Fig. 7. The visualization improvement of the blood vessels is not much [as shown in Figs. 7(b) and 7(c)] when the weight of C-scan noise (i.e., β) is small. On the contrary, if the weight of the C-scan noise is too large, some blood flow signals could be considered noise to be removed, resulting in poor vascular continuity and density, as shown in Fig. 7(e). Among these results, Fig. 7(d) demonstrates the best improvement of vascular visualization with the proper balance of C-scan noise and single-shot *en face* image in image synthesis. Therefore, we choose to set α=0.25 and β=0.75 to achieve superior image enhancement in our deep learning model.

### 3.2 Enhancement of OCTA Images by Proposed Deep Learning Method

**Fig. 8:**
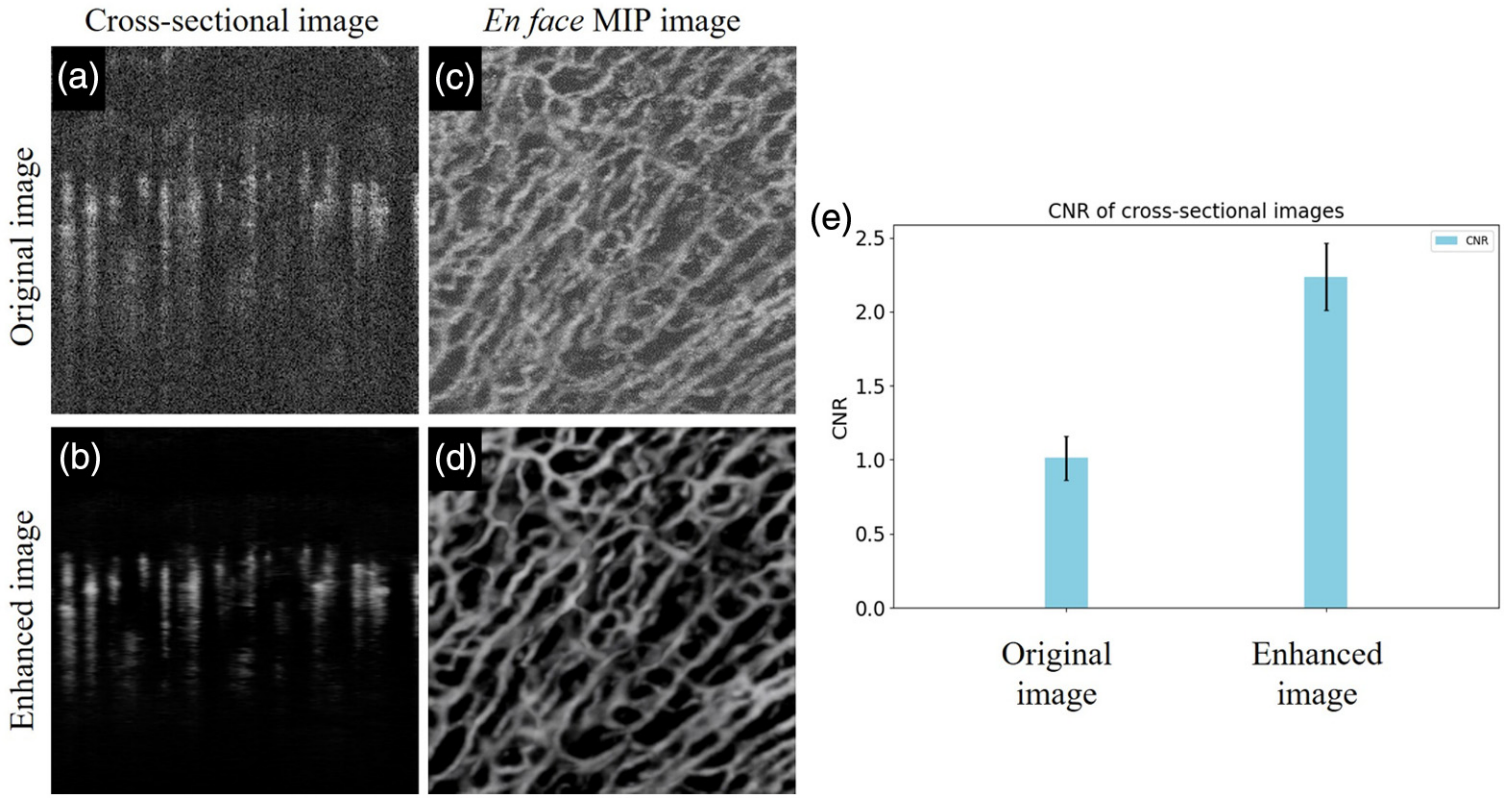
(a) and (b) Cross-sectional OCTA images before and after image enhancement, respectively. (c) and (d) *En face* MIP OCTA images before and after image enhancement, respectively. (e) CNR of the cross-sectional images.

We demonstrate several enhancement results of OCTA images to verify the superior performance of our proposed method. Figure 8 shows the cross-sectional and *en face* MIP images before and after the image enhancement. In the original cross-sectional OCTA image [Fig. 8(a)], considerable speckle noise can be observed, resulting in poor visualization of blood vessels in the *en face* MIP image [Fig. 8(c)]. After our deep learning enhancement of the 3D OCTA images depth-by-depth in the C-scan direction, we can reconstruct quite a clean cross-sectional OCTA image [Fig. 8(b)]. Here, we performed CNR quantification analysis on the cross-sectional images to quantify the denoising effect. The area above the dermal layer, which does not contain blood vessels, is selected as the background region of CNR. An adaptive threshold method is utilized to binarize the cross-sectional image, and the area of white pixels is considered the signal region. After calculating the CNR of 256 cross-sectional images, we plotted the column chart with an error bar, as shown in Fig. 8(e). The CNR of enhanced images is ∼2 times better than the original images. Therefore, the enhanced *en face* OCTA image [Fig. 8(d)] has much better visualization with less background noise and a clearer vascular network than the original one.

**Fig. 9:**
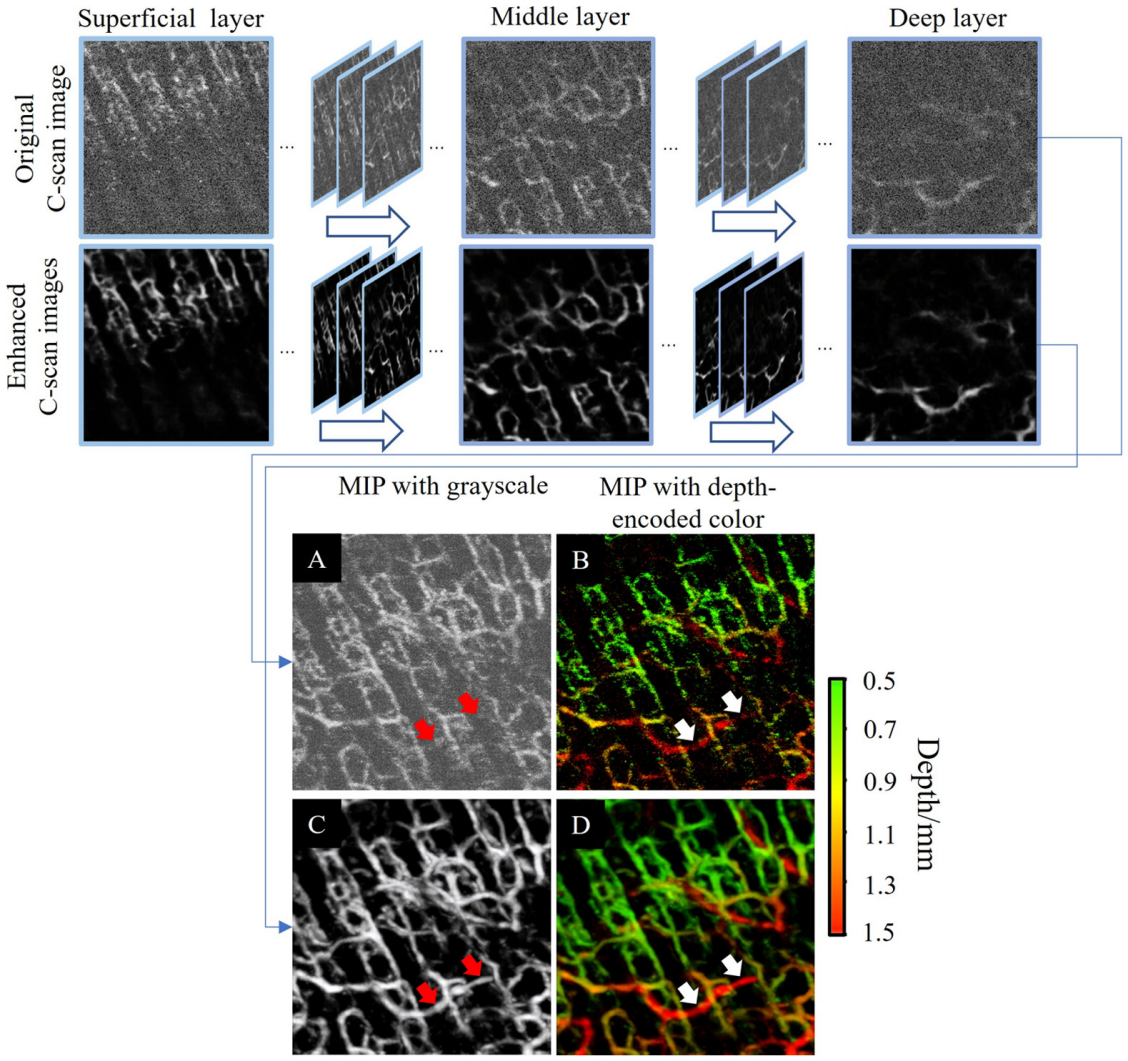
Comparison of OCTA image enhancement with depth information. (a) and (b) Original *en face* MIP with grayscale and depth-encoded color, respectively. (c) and (d) Enhanced *en face* MIP image with grayscale and depth-encoded color, respectively. MIP, maximum intensity projection.

Figure 9 shows more details of the OCTA image enhancement with depth information from the superficial to a deep layer of skin tissue. The quality of the original C-scan images greatly suffers from speckle noise, where the profiles of blood vessels are not very smooth. When it goes into the deeper layer of skin, the blood vessel signal is very weak, resulting in the deep vascular network being barely visualized. With proper training, our deep learning model can obtain the pattern feature of the vascular network, remove the speckle noise, and greatly enhance the blood vessel signal. Figures 9(a) and 9(c) are the grayscale *en face* MIPs of the 3D OCTA images before and after image enhancement. The enhanced MIP has much better CNR and smoother vessel profiles than the original one. Moreover, as pointed out by the red arrows, our deep learning method greatly improved the visualization of some blood vessels that might have been considered background noise in the original MIP. We encode the depth information with the MIP using a green-to-red color bar, as shown in Figs. 9(b) and 9(d). The blood vessels pointed by the white arrows are red, which means they are located in the deep dermal layer of the skin. The comparison of MIPs with depth-encoded color demonstrates that our proposed method can significantly improve the visualization of blood vessels in the deep layer and provide a more comprehensive vascular network in biological tissue.

**Fig. 10:**
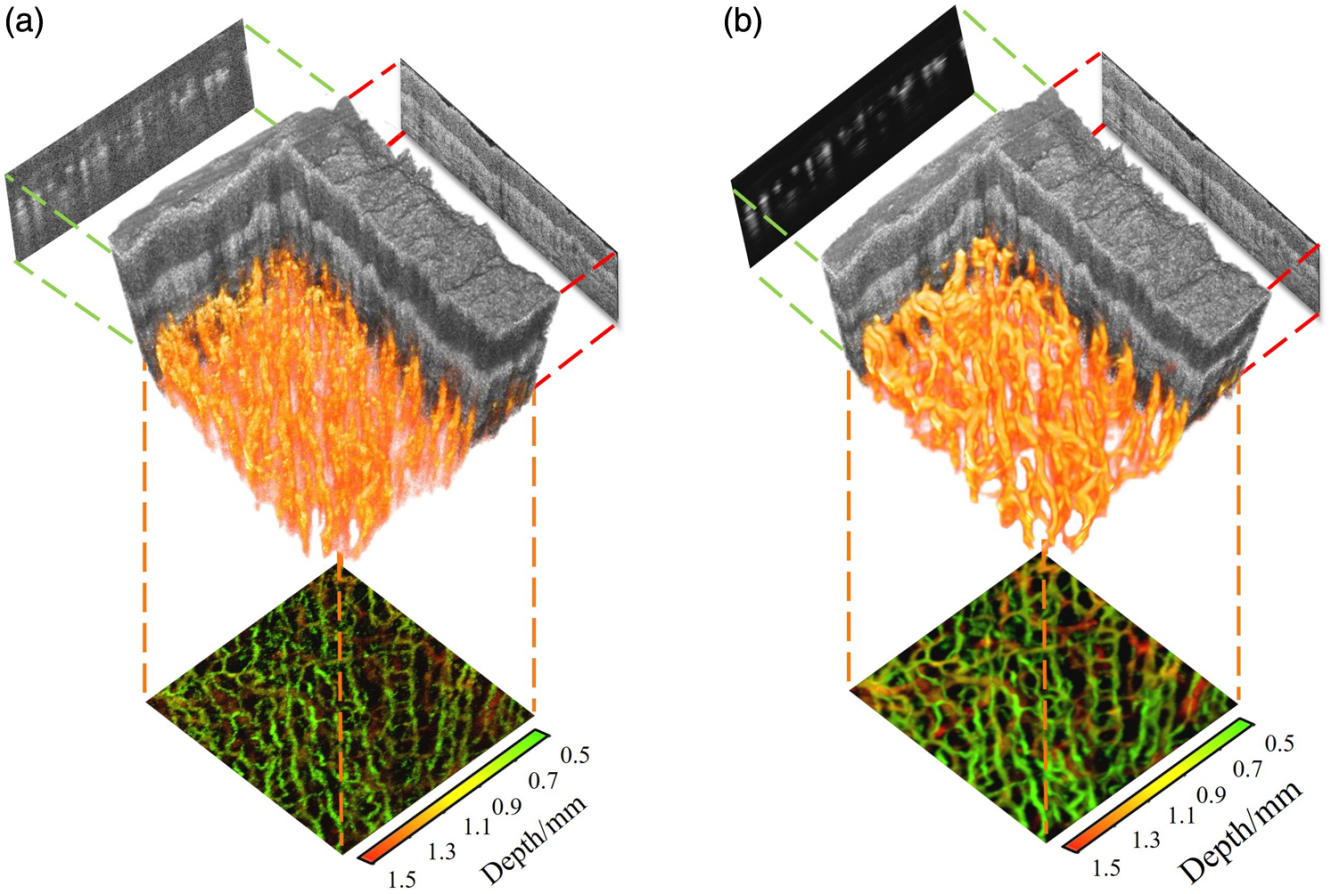
Volume renderings before (a) and after (b) image enhancement.

Figure 10 shows the volume renderings of 3D OCT and OCTA images before and after image enhancement. The structural OCT information helps us understand the position and distribution of the blood vessels in the skin. The volume renderings verify that there are no blood vessels in the epidermal layer and there are plenty of vascular plexus in human palm skin to provide nutrient supply. However, because the original OCTA images have too much speckle noise, the volume rendering of blood vessels is very fuzzy, making it difficult to identify the vascular network. On the contrary, our deep learning method has great denoising performance, providing a clear visualization of blood vessel topology in enhanced volume rendering.

### 3.3 Comparison of OCTA Image Enhancement

**Fig. 11:**
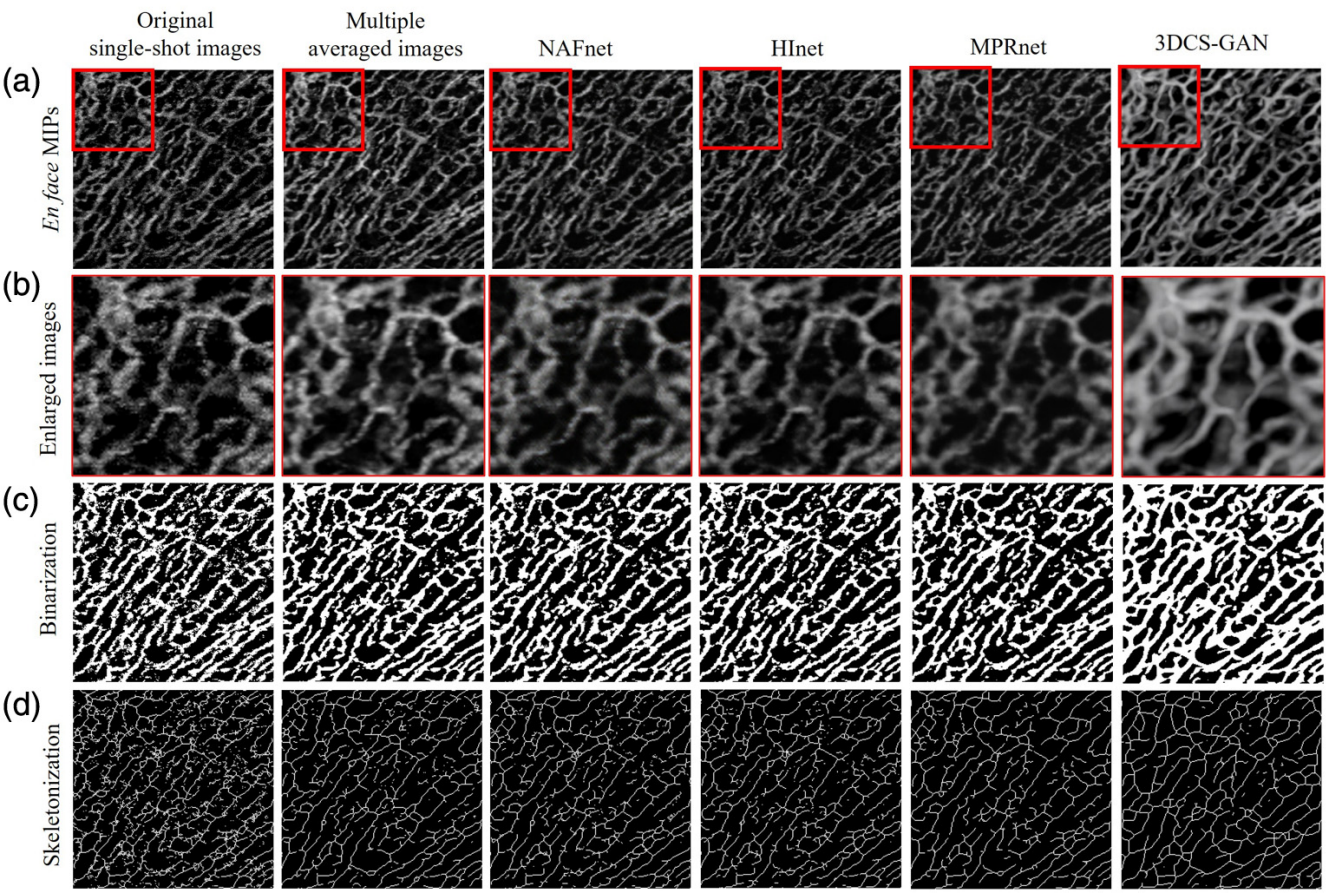
Visual comparison of original single-shot, multiple averaged, and enhanced images by different deep learning methods. (a) *en face* MIPs. (b) Enlarged images of the region indicated by the red square box. (c) Binarized images. (d) Skeleton maps.

We further conduct a quantitative evaluation of the *en face* OCTA images to emphasize the advantage of our proposed method. Figure 11 shows representative original single-shot *en face* image, multiple averaged *en face* image, and an *en face* image enhanced by other state-of-the-art (SOTA) methods, *en face* image enhanced by the proposed method, and key processing results for quantitative evaluation. In this study, we choose nonlinear activation free network (NAFnet),^43^ half instance normalization network (HInet),^44^ and multipath residual network (MPRnet)^45^ as other SOTA methods for comparison. These SOTA methods have demonstrated effective handling of complex noise in image denoising tasks while preserving image details. These SOTA methods were trained using multiple averaged *en face* images as the training target and single-shot scans as input. They were directly trained on the *en face* images and compared with our method of depth-by-depth enhancement. Figures 11(a) and 11(b) visually compare these OCTA images and demonstrate their intuitive difference. The multiple averaged images had a smoother vascular profile and less background noise than the original single-shot images. In addition, our enhanced images through our method exhibit the best vessel visualization compared with other deep learning approaches trained directly on *en face* images, with relatively smooth contours and uniform intensity. To quantitatively evaluate these images, we binarized them by a global adaptive threshold method, as shown in Fig. 11(c). The CNR of the *en face* OCTA images was calculated by selecting the black areas of the binarized image as the background region and the white areas as the signal region according to Eq. (5). The VD was also easily calculated using Eq. (6). Then, we skeletonized the binarized images to obtain the vascular skeleton map, as shown in Fig. 11(d). Many fake branches and spikes can be seen in the skeleton map due to the low image quality. Finally, we calculated the evaluation metrics of VDI and VC according to Eqs. (7) and (8). The quantitative comparison of data sets of images is presented in Table 1. The results indicate that the enhanced images generated by a three-dimensional C-scan-based generation adversarial network (3DCS-GAN) have the best image quality in terms of CNR, VD, VDI, and VC, confirming the superior performance of our proposed method. To further validate the superiority of 3DCS-GAN, we performed independent sample t-tests on metrics such as CNR, VD, VDI, and VC (significance level p<0.01). The results showed that 3DCS-GAN significantly outperformed other methods in terms of VD, VDI, and VC metrics (Table 1).

**Table 1:** Quantitative evaluation of the OCTA images (mean ± *SD*).

	Original single-shot images	Multiple averaged images	NAFnet	HInet	MPRnet	3DCS-GAN
CNR	1.640 ± 0.130	2.021 ± 0.101	2.001 ± 0.009	2.040 ± 0.091	1.978 ± 0.074	**2.082 ± 0.120**
VD	0.333 ± 0.016	0.347 ± 0.008	0.336 ± 0.015	0.332 ± 0.014	0.345 ± 0.015	**0.448 ± 0.044** ^**^
VDI	2.743 ± 0.218	4.350 ± 0.125	4.106 ± 0.585	4.207 ± 0.103	4.720 ± 0.120	**5.179 ± 0.104** ^**^
VC	0.919 ± 0.014	0.967 ± 0.005	0.958 ± 0.007	0.956 ± 0.004	0.968 ± 0.008	**0.986 ± 0.003** ^**^

Note: The bold numbers represent the best evaluation metrics. SD, standard deviation; CNR, contrast-to-noise ratio; VD, vessel density; VDI, vessel diameter index; and VC, vascular continuity.

** Indicates p<0.01 (compared with all comparison methods).

### 3.4 Evaluation of 3DCS-GAN for OCTA Images of PWS Disease

**Fig. 12:**
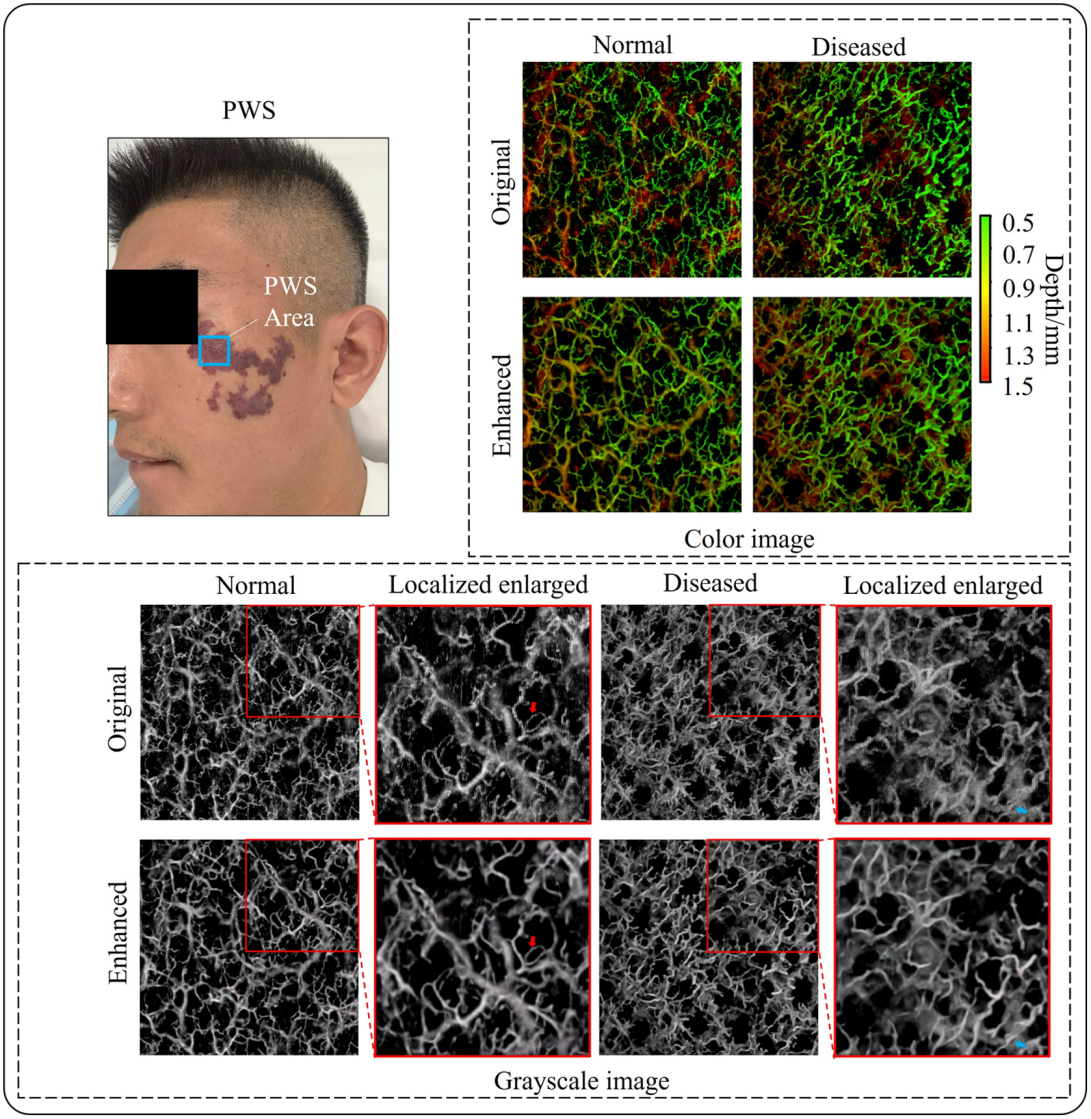
Visual comparison of PWS OCTA images using the 3DCS-GAN method. The white scale bar is 0.5 mm. The FOV is $6\times 6\;{\mathrm{mm}}^{2}$.

After analyzing the test results for normal skin, we further verify the effectiveness of the 3DCS-GAN method in enhancing skin disease images by applying it to OCTA images of PWS disease. Patients with PWS were recruited from the Department of Laser Medicine, the First Medical Center, Chinese PLA General Hospital. This study was approved by the Ethics Committee of the Chinese PLA General Hospital, and informed written consent was obtained from each patient. The results are shown in Fig. 12, which include the OCTA images of PWS disease and contralateral normal skin. We locally magnified the grayscale OCTA images, as indicated by the red box to show the details. In the magnified images, it is evident that the enhanced images are generally smoother and more uniform than the original images, with a more pronounced vascular structure and improved connectivity, as indicated by the red arrows. The enhanced images also allow us to more easily discern the distinction between the superficial and deep blood vessels, as denoted by the blue arrows.

To further quantify the enhancement effect of the proposed method on skin disease OCTA images, we used the evaluation indicators VC, VD, and VDI to perform a quantitative analysis of the vascular information in the normal and diseased images, as shown in Table 2. The results show that the vascular density of the diseased images with PWS is higher than that of the normal images. For the OCTA images of normal skin tissue, the vascular distribution is relatively sparse, and the vascular edges become blurred and difficult to be accurately segmented due to the influence of noise. Some vascular points are filtered out as noise points, leading to a decrease in vascular density. However, the enhanced images have reduced these effects, resulting in a higher vascular density. For the OCTA images of diseased skin tissue, the blood vessels are inherently rich and dense. Noise may be erroneously recognized as more vascular structures, leading to a higher vascular density compared with the enhanced images. In summary, the 3DCS-GAN method has achieved good results in enhancing skin disease OCTA images, allowing for better presentation of the vascular structure and depth information.

**Table 2:** Quantitative evaluation of blood vessels in OCTA images.

	Normal		Diseased	
	Original	Enhanced	Original	Enhanced
VD	0.351	0.368	0.435	0.405
VDI	3.018	5.297	3.646	5.547
VC	0.911	0.976	0.966	0.987

Note: VD, vessel density; VDI, vessel diameter index; and VC, vascular continuity.

**Fig. 13:**
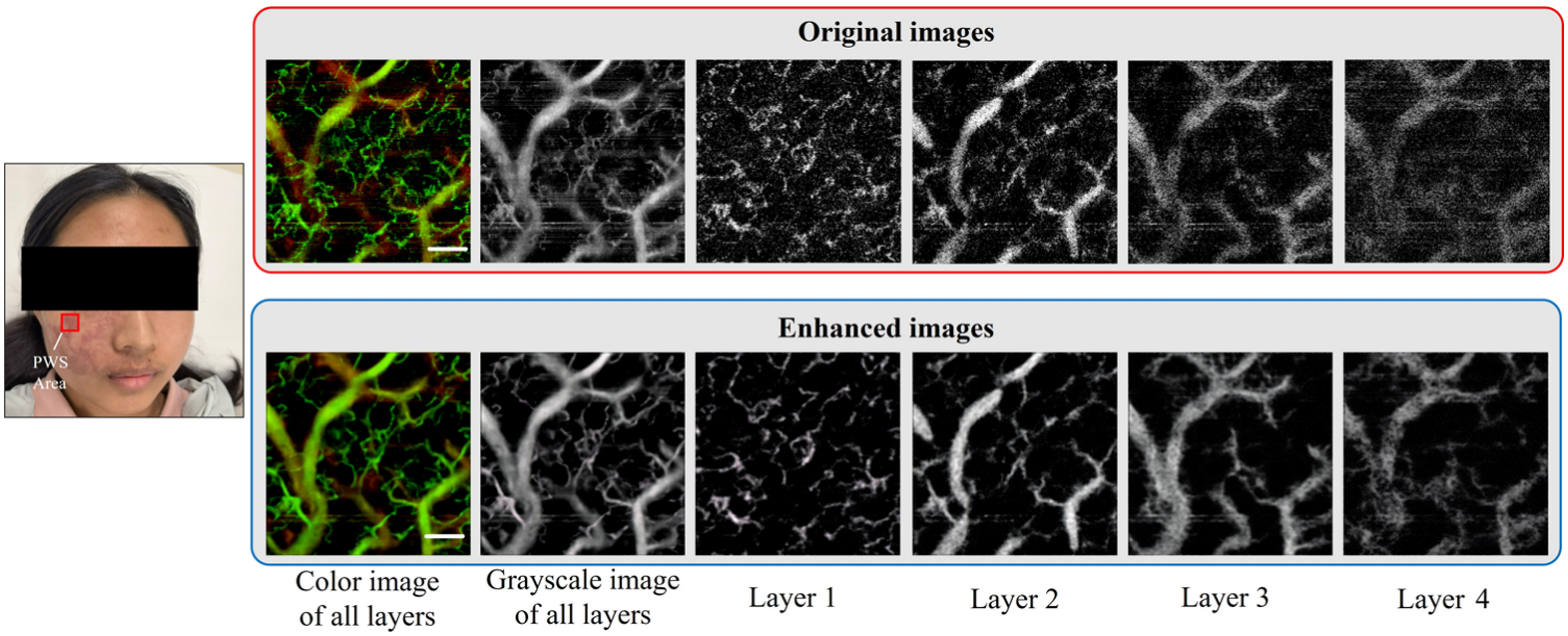
OCTA deep slice display of PWS disease. The white scale bar is 0.5 mm. The FOV is $3\times 3\;{\mathrm{mm}}^{2}$.

The depth information of blood vessels is one of the most critical factors influencing the therapeutic outcome in PWS. OCTA is a noninvasive tool for observing the depth of malformed blood vessels in PWS. Figure 13 shows the OCTA images of another patient who is diagnosed as red-type PWS. The *en face* images of all layers provide comprehensive vascular information on PWS. The OCTA volume in the dermal layer was segmented and divided into four layers, where each layer has a thickness of ∼0.3  mm. As the imaging depth increases, the light scattering and absorption effects within the biological tissues lead to a significant attenuation of the vascular signal intensity. The original OCTA images have a lot of speckle noise, and the ectatic blood vessels in the deep dermis (i.e., layers 3 and 4) are quite weak and noisy. The influence of background noise and weak vascular signal makes accurate identification and quantitative analysis of deep blood vessels in PWS extremely difficult. As shown in the bottom images of Fig. 13, the 3DCS-GAN algorithm greatly enhances the vascular signal while suppressing the background noise. The proposed method clearly reconstructs the structure of deep blood vessels, improving the image’s interpretability and diagnostic value.

## 4 Discussion and Conclusion

The use of OCTA in biomedical applications has increased significantly in recent years, and thus, generating high-quality images of the vasculature is very important for clinical diagnosis and biological research. This article presented a new deep learning–based processing method that fully utilizes volumetric OCTA data for image quality enhancement. Compared with previous deep learning studies, the proposed method has two major innovations. First, previous deep learning methods only perform their algorithms on the B-scan frames or the final *en face* OCTA projections. By contrast, the proposed method applies the deep learning algorithm on the C-scan image depth-by-depth, enabling it to achieve superior performance in OCTA image enhancement. Second, images with avascular C-scan noise images are used to synthesize the input data for network training. The parameters α and β determine the weight ratio between blood flow images and C-scan noise images. This avoids the time-consuming and complicated work of obtaining high-quality C-scan OCTA images as reference labels. The training strategy by synthetic input helps our model learn the topological feature of the vascular network in *en face* view, greatly reducing the speckle noise for C-scan noise. It is important to note that the weight ratio is based on experiments and observations rather than derived from clinical or physical principles. Future studies are needed to establish a more rigorous and clinically meaningful method for determining these parameters.

It should be noted that using multiple B-scans for registration presents challenges, particularly regarding alignment accuracy and subject motion. Even slight movement in the slow scanning direction can shift B-scans, making registration difficult and reducing image quality, which in turn makes it less reliable for obtaining ground truth labels for training. In addition, methods that extract dynamic signals from multiple B-scan acquisitions at the same position require the subject to remain still for an extended period, which is impractical. By contrast, the registration of *en face* OCTA images offers a key advantage: misaligned sections can simply be cropped out, preserving only well-aligned areas. Our approach enhances robustness against motion artifacts while maintaining efficiency, providing a more practical method for generating high-quality ground truth data. Although the deep learning architecture and training methodology in this study are not particularly unique, the primary focus of our work is on improving the quality of training data and applying the deep learning algorithm to C-scan images depth-by-depth. Our approach provides a more robust solution for generating high-quality OCTA images, which cannot be achieved simply using current SOTA methods.

Our main objective is to illustrate that utilizing deep learning techniques for the construction of *en face* OCTA images from enhanced C-scan images can more effectively preserve the topological features of three-dimensional vascular networks compared with the deep learning methods that directly enhance *en face* OCTA images. In our study, the 3DCS-GAN method adopts the architecture of Pix2Pix, which incorporates a specially designed U-Net generator optimized for image-to-image translation, whereas simple conditional GAN may be less optimized for detailed image mapping. In addition, other SOTA deep learning methods, such as NAFnet, HInet, and MPRnet, are employed to directly improve *en face* OCTA images. It is important to note that these SOTA methods are neither trained nor process images in the same manner as our proposed method, which could lead to an unfair comparison. However, the primary focus of our research is not to compare different deep learning architectures but rather to introduce a novel strategy for synthesizing training data and implementing depth-by-depth deep learning processing. This approach yields superior results in terms of preserving critical vascular information, thereby offering a significant advancement in the field of OCTA.

In conclusion, we propose a superior deep learning method called 3DCS-GAN to enhance the vascular visualization for OCTA images. 3DCS-GAN is advantageous because it obtains the topological feature of the vascular network from the *en face* OCTA image and performs depth-wise denoising on the volumetric OCTA data. The synthesis data set greatly reduces the laborious work of obtaining high-quality reference labels for network training. The qualitative and quantitative evaluations verify the superiority of 3DCS-GAN for OCTA image enhancement. In the case of PWS disease, 3DCS-GAN is able to greatly reduce the background noise and improve the visualization of deep-layer vasculature in OCTA, which helps physicians to more comprehensively evaluate the condition of PWS patients. The proposed method is promising for use in the reconstruction of high-quality 3D vasculature in clinical diagnosis without increasing system complexity and data acquisition time.

## Data Availability

All relevant code, data, and materials are available from the authors upon reasonable request. Correspondence and requests should be addressed to the corresponding author.
